# Clinical Cases in the Medical Wards of the Mercy Hospital

**Published:** 1872-11-15

**Authors:** N. S. Davis


### Clinical Reports.

### CLINICAL CASES IN THE MEDICAL WARDS OF THE MERCY HOSPITAL.

SERVICE OF PROF. N. S. DAVIS.

*Reported by D., Nov.* 1, 1872.

*Typho-Malarial Fever:* The patient was a male, aged 23 years, who had had several paroxyms of intermittent fever during the latter part of August and the month of September, in consequence of which he had become pale and somewhat debilitated, but was recovering until about two weeks since. He was then attacked with a chill, followed by a paroxysm of fever, which abated after a few hours, but has not since entirely disappeared. He says he took some quinine and other medicines, but instead of having the progress of the fever interrupted, it was only modified in its progress. There were no distinct repetitions of the chill, but a paleness and sense of weakness each day, during the morning hours, with increased heat, quickness of pulse, headache, restlessness, and general febrile symptoms during the afternoon and evening; and in all parts of the day and night, a swimming, unsteady feeling in the head when in the upright position; dryness of the lips and mouth; tongue covered with a dirty, white coat; pulse 100 per minute; abdomen moderately full and tympanitic ; bowels relaxed; and the mind dull. He had no appetite, and only moderate thirst. The professor remarked that the patient had been in the hospital only four or five days, and was affected by a mixed grade of fever. The early history of the case, together with the existing paleness of skin, and exacerbating character of the febrile phenomena, show the existence of a malarious influence; while the dullness of mind, dryness of lips and mouth, sense of giddiness in the head, together with symptoms of diarrhœa and abdominal tympanites, clearly indicate a typhoid condition and tendency. In other words, the case has become one of typho-malarial fever, of moderate severity. It was remarked that this mixed grade of fever had been very prevalent in the city from the early part of August to the present time. While in the surrounding country districts purely malarious or periodical fevers have been unusually prevalent. Many of the typho-malarial cases in the city have commenced with such distinct chills and exascerbations of fever, as to lead the patient and practitioner both to regard them as simple malarious cases, and to confidently expect their full interruption by antiperiodics. In a few, the deception has been increased by an interruption of the paroxysms for two or three days after the first induction of the effects of quinine so fully that the patients have gotten up, and supposed themselves convalescent. Yet there has remained with them a dull, unnatural feeling in the head; a dry or gummy feeling in the mouth; obscure achings in the back and limbs, especially in the afternoon, with indifference to food; and after a period ranging from three to five days, the face would become suffused with redness; the lips dry; the tongue coated; more dullness, swiming and pain in the head; and more general derangement of secretions. The pulse from 100 to 110, and temperature from 100° to 103° in the afternoon, but so much less as to constitute a remission in the morning. From this time on, every day generally adds to the predominence of the typhoid symptoms, and some of the cases run a protracted course of from four to six weeks. If, when the fever returns, after the first apparent interruption by quinine, that remedy be again resorted to, and the doses increased, with the expectation of again arresting, at once, the further progress of the case, it generally produces no other effect than to stupify the sensibility of the patient, and add to the giddiness and confusion in the head. The method of treatment which the professor has found most beneficial in these cases, when called to them in the early stage, has been, rest; a bland, simple diet; milk-whey for drink; sponging the surface with luke-warm water, when hot and dry in the afternoon; and the exhibition of a mixture of carbolic acid, gelsemin, and camphorated tincture of opium, in moderate doses, to counteract the typhoid elements, and moderate anti-periodic doses of quinine in the morning remission to destroy malarious influence. Four or five days since, when the present patient was admitted to the hospital, he was ordered the following prescriptions:

℞ Carbolic acid crystals, 6 grs.

Glycerine (pure), ℥ ss.

Tinct. gelsemin, ʒ iii.

Camph. tinct. opii., ℥ iss.

Aqua, ℥ iss.

Mix. Give one teaspoonful every four hours.

℞ Sulph. quinine, 24 grs.
Hydg. chlorid. mite., 3 grs.
Pulv. liquorice root, 6 grs.

Mix. Divide into six powders, and give one at six and ten o'clock each morning.

He has continued these medicines steadily until the present time, and with a gradual improvement in all the febrile symptoms. He was directed to continue the carbolic acid solution every six hours, and to take only one powder of the quinine each morning. The

Professor remarked that this case would probably require no other treatment except the gradual withdrawal of his present medicines, as convalescence became established, and the allowance of a more liberal diet.

When cases of this mixed fever assume greater severity, and, after the second week, present more prominent typhoid symptoms, the treatment must be more fully that which is adapted to the advanced stage of idiopathic typhoid fever.

*Progressive Locomotor Ataxy, following Malarious Fever:* This patient is a male, about 22 years of age ; laborer ; and native of Ireland. This history showed that he had been attacked with intermittent fever during the latter part of August, which was interrupted by antiperiodics, but returned at irregular intervals up to the time of his admission into the hospital, about one week since.

Seeing him first in bed, and learning that he had been affected with chronic ague, he was ordered moderate doses of quinine and iron, with simple but nourishing diet. Three days passed without the recurrence of chills or fever, but at each visit he complained of an unusual giddiness in his head, and what he called weakness in his limbs.

On scrutinizing more closely the past history and present condition of the patient, it was ascertained that the feeling of unsteadiness in his head, and a gradually increasing difficulty in walking, had existed during the last three or four weeks. The Professor first called the attention of the class to the general aspect of the patient, and then to his mode of walking. He was pale, or anemic; moderately emaciated; pulse 85 per minute; respiration natural; tongue clean; moderate appetite; bowels nearly natural; but a con stant dull pain and sense of swimming in the head. On attempting to walk the length of the ward, his head was poised in a certain position, his step hesitating and unsteady, his gait reeling or staggering, in spite of his efforts to the contrary, and his getting up and sitting down peculiarly careful and unsteady. He could not get up from his chair quick, or walk far without danger of falling.

The symptoms were closely analagous to those of progressive locomotor ataxy, more especially as regarded the muscular movements. There was present, however, no marked alterations of sensibility in any part of the cutaneous surface, nor derangements of vision. In regard to the special pathology of the case, it was remarked that the patient's blood had become spanemic or impoverished by the protracted influence of malaria ; and this, acting in conjunction with other less appreciable causes, had led to the impairment of the nutrition of the spinal cord, and its junction with the crura cerebri and cerebelli, thereby inducing both the unpleasant feeling in the head and the impaired co-ordination of muscular movements. The case might be regarded, therefore, as one of incipient progressive locomotor ataxy, but not so far advanced as to preclude the idea of recovery. The indications for treatment were stated briefly, to consist in efforts to restore a healthier condition of the blood, by increasing its nutritive elements and red corpuscles, and in exciting a more active nutrition of the cerebro-spinal nervous system. The best means for fulfilling these indications, so far as the Professor's experience can be relied on, are, good air; a nutritious but easily digested diet, consisting largely of light bread and milk tenderly cooked meats, and fresh vegetables very moderate exercise, always followed by rest, in the recumbent position; and the persistent use, internally, of the phosphatic tonics, of which the best is a mixture of the extract of malt, two parts, with one of the compound syrup of hypophosphites, given in doses of from two to three drachms, at each meal time. If the patient is free from any influence of malaria, and his bowels regular, he will not be benefitted by any additional medicine. But if, as in the present case, the patient has had recent paroxysms of intermittent fever, it will be desirable to give a pill of two or three grains of quinine every morning. Or if the bowels are costive, a pill containing one grain each of extract of hyosciamus, sulphate of iron, and pulverized gum aloes, may be given at bed time. The present case was put on the use of the extract of malt and compound syrup of hypophosphites, with one moderate dose of quinine each morning. A few days since, the attention of the class had been called to a case presenting many of the symptoms of spinal atrophy, complicated with occasional paroxysms of epilepsy. He was put on the treatment here indicated, with the addition of one dose of twenty grains of the bromide of potassa at bed time each night.

Another case was alluded to, which had been treated in the hospital about one year since, and in which all the characteristic symptoms of progressive locomotor ataxy were strongly developed. The power of coordination of muscular movements was so impaired that for several weeks the patient could not walk or maintain the upright position without assistance. He was treated with quinine, iron and strychnine for many weeks, aided by good food, frictions, and some use of electro-magnetism, but with very little improvement. He was then put on the use of the extract of malt and hypophosphites, plain, nutritious food, and allowed to rest. After some time he began to gain gradually, and in about four months had so far recovered that he walked well, and left the hospital in fair health.

---

Poisoning by Phosphorus; Cure by Turpentine, by Dr. Vito Giuseppe, of Marco.—The case was that of a woman aged 40, who had swallowed, fasting, with the intention of suicide, an infusion in which a package of ninety matches had been placed. The author, called two hours after ingestion of the poison, observed the following symptoms: Pain, most intense at the epigastrium, facies abdominalis, filiform pulse, respiration very frequent, etc. He administered an emetic at once, then a gramme (15 grs.) of essence of turpentine. The sypmptoms soon amended but the patient was seized with a severe diarrhœa and passed a large quantity of urine. In two days she had recovered.

The author thinks that the turpentine acted in this case as a diuretic and purgative in favoring the elimination of the phosphorus; he rejects the theory of Personne (preventive to the combustion of phosphorus).—*Il Morgagni Giornale No.* 34, 1872.—*The Clinic.*

It is said that 180 German-educated physicians arrive monthly in New York.

Advertising Specialists.—The Michigan State Medical Society has adopted a resolution favoring the insertion of the advertisement of the cards of physicians and specialists in the secular papers. Although nothing more is allowed than the mention of the name, specialty, and office address, we fail to see how the public can, in the majority of instances, draw the line between legitimate medicine and quackery. This provision is evidently for the benefit of the specialists in the State, and especially for such as have not proved their competency to their legitimate judges—the profession at large. Physicians, as a class, are discriminative enough to judge whether or not a specialist, so-called, is to be trusted with their patients, and sensible patients are very apt to ask their family medical advisers before consulting strangers. It is by such recommendations that specialism has thriven, and competent practitioners in in any particular branch have never wanted patients when they have proved themselves worthy of them. If a specialist wishes to advertise himself as such, let him convince the profession of his qualifications by suitable contributions to medical journals, and by original investigations in his particular branch. If we step aside from this rule, and endorse such brazen individuals who can advertise themselves in no other way than by the insertion of their cards in the medical journals or secular papers, we rob legitimate specialism of its well-deserved laurels, and encourage every misguided and presumptuous pretender who may desire to trade upon our charity, or upon the public's credulity.—*Boston Medical and Surgical Journal.*

Anchylosis of the Inferior Maxilla Cured by the Formation of a False Joint on each Side (G. Maas: *Arch. f. Klin. Chirurgie*, xiii., B. 3, H.). — A man, 27 years old, had had in his seventh year an attack of scarlatina, which left him with an inflammation of the maxillary articulation on both sides, which was followed by complete anchylosis. Both the inferior and superior maxillary bones had suffered in consequence an arrest of development. Middledorpf exercised on one side, and subsequenely Fischer on the other, a wedge-shaped piece from the inferior maxilla, the base of the wedge being downwards, according to Esmarch. The mobility had remained perfect at the time of the report, four months after the operation. — *Phil. Times*, Oct. 12, '72.

Wiesbaden has been selected as the seat of the next session of the German Medical Congress.

